# Declining rates of sterilisation reversal procedures in western Australian women from 1990 to 2008: the relationship with age, hospital type and government policy changes

**DOI:** 10.1186/s12905-017-0470-3

**Published:** 2017-11-25

**Authors:** Khadra A. Jama-Alol, Alexandra P. Bremner, Gavin Pereira, Louise M. Stewart, Eva Malacova, Rachael Moorin, David B. Preen

**Affiliations:** 1 Centre for Health Services Research, School of Population Health, The University of Western Australia, 35 Stirling Highway, Crawley, WA 6009 Australia; 2 School of Population Health, The University of Western Australia, 35 Stirling Highway, Crawley, WA 6009 Australia; 3 School of Public Health, Curtin University, Kent Street, Bentley, WA 6102 Australia; 4 Centre for Population Health Research, Faculty of Health Sciences, Curtin University, Kent Street, Bentley, WA 6102 Australia

**Keywords:** Sterilisation reversals, Private health insurance, Lifetime health cover, Medicare levy surcharge, Linked population health data

## Abstract

**Background:**

Female sterilisation is usually performed on an elective basis at perceived family completion, however, around 1–3% of women who have undergone sterilisation elect to undergo sterilisation reversal (SR) at a later stage. The trends in SR rates in Western Australia (WA), proportions of SR procedures between hospital types (public and private), and the effects of Federal Government policies on these trends are unknown.

**Methods:**

Using records from statutory state-wide data collections of hospital separations and births, we conducted a retrospective descriptive study of all women aged 15–49 years who underwent a SR procedure during the period 1st January 1990 to 31st December 2008 (*n* = 1868 procedures).

**Results:**

From 1991 to 2007 the annual incidence rate of SR procedures per 10,000 women declined from 47.0 to 3.6. Logistic regression modelling showed that from 1997 to 2001 the odds of women undergoing SR in a private hospital as opposed to all other hospitals were 1.39 times higher (95% CI 1.07–1.81) and 7.51 times higher (95% CI 5.46–10.31) from 2002 to 2008. There were significant decreases in SR rates overall and among different age groups after the Federal Government interventions.

**Conclusion:**

Rates of SR procedures in WA have declined from 1990 to 2008, particularly following policy changes such as the introduction of private health insurance (PHI) policies. This suggests decisions to undergo SR may be influenced by Federal Government interventions.

## Background

Female sterilisation is a common birth control method [1], although rates have declined in Australia and internationally in recent times [2, 3], and it is usually performed on an elective basis at perceived family completion [4]. Around 1–3% of women who have undergone sterilisation elect to undergo sterilisation reversal (SR) or anastomosis at a later stage [5–7]. Requests for renewed fertility have been linked to changes in marital status, desire for more children, death of a child and improved economic status [8–10].

Curtis et al. (2006) reported that, compared with women who undergo sterilisation when they are older, women who are sterilised prior to 30 years of age are twice as likely to regret their decision and 8-times more likely to request information about SR procedures, undergo SR or have an evaluation for in vitro fertilisation (IVF) than women over 30 years [11]. Current options for renewed fertility include SR which, when successful, offers the prospect of spontaneous pregnancy comparable with that of natural (unassisted) pregnancy rates in the community [8]. Other options such as IVF are also available for restoring fertility, and these assisted reproductive technologies have become more accessible over recent decades [12].

Health care funding and subsidies can affect the medical procedures that people choose. In Australia, health care is funded on a dual basis. All Australian citizens and permanent residents are eligible under the taxpayer-funded Medicare system to subsidised primary and secondary care [13–15]. In addition, many Australians purchase personal or family private health insurance (PHI). In the last two decades, the Federal Government has introduced a number of policies to encourage the uptake of PHI [15, 16], with the intention of reducing the taxpayer burden on funding the public system.

Since the withdrawal of the Medicare subsidy for SR procedures in 1997, the choice has been to either self-fund SR operations or to undergo Medicare-funded IVF [8]. In 1997 the Medicare Levy Surcharge, a tax penalty of 1% of taxable income payable by individuals in higher income brackets without PHI (taxable incomes in excess of $70,000 per year for single individuals and $140,000 per year for couples) [16, 17] was introduced, which increased PHI uptake nationally [16]. In addition, in 2000, a Lifetime Health Cover (an incremental age-based penalty imposed on individuals who first purchase PHI after age 30 years) was introduced to increase PHI uptake rates [16].

IVF has been available in Australia since 1980 [18], but subsidised through Medicare since 2001 [12], and subsequently it has become the preferred choice for restoring fertility [19]. On 1st July, 2004, the Australian Federal Government introduced a ‘Baby Bonus’ scheme under which mothers received $3000 per new child, increased to $4000 on 1st July 2006 and to $5000 on 1st July 2008 [20, 21].

There is limited information about the incidence of SR in Australia and the impact of Federal Government policy changes on rates of SR. We used whole-population linked administrative data to explore trends in SR among Western Australian (WA) women of reproductive age (15–49 years), by age group and hospital type (public or private). We also evaluated the influence of the Federal Government interventions, such as the withdrawal of the Medicare subsidy for SR procedures and the introduction of the Medicare Levy Surcharge and Lifetime Health Cover policies that were implemented to increase uptake of PHI, on SR rates during the study period.

## Methods

### Study population

The study sample included all females of child bearing age (defined as 15–49 years, according to previously established methods) [22, 23], who were resident in WA and had a record in the WA Hospital Morbidity Data Collection (HMDC) of having undergone a SR procedure from 1st January 1990 to 31st December 2008. SR inpatients were identified from the principal and up to 10 secondary procedure codes, as well as from principal diagnosis and up to 20 secondary diagnoses codes on each record in the HMDC. SR was identified based on contemporaneous versions of the International Classification of Disease version 10 with Australian modifications (ICD-10-AM) and included [35694–01 (laparoscopic anastomosis of fallopian tube), 35,694–05 (anastomosis of fallopian tube), 35,697–00 (microsurgical laparoscopic anastomosis of fallopian tube) and Z31.0 (tuboplasty), ICD-9-CM: V26.0 (tuboplasty)] [24–26].

### Data sources

#### Hospital morbidity data collection and midwives notification system

Study data were extracted from two statutory state-wide data collections: the HMDC, which includes routinely-collected data on all discharges from all public and private hospitals in WA (in WA SR is performed in hospitals), and the Midwives Notification System (MNS) which routinely collects records for all births in WA. Data from these collections were linked through the WA Data Linkage System (WADLS) using validated and best-practice probabilistic matching techniques as previously described [27]. Dates for all episodes of SR as well as woman’s date of birth, country of birth, Indigenous status, and the hospital type where the procedure occurred were obtained from the HMDC. Data on previous pregnancies and the date of birth of each resulting child were obtained from the MNS to determine parity prior to the first hospital admission for an SR procedure.

To determine appropriate denominators of women ‘at risk’ for SR, data on the population of women who had been sterilised in WA were extracted from HMDC, as described by Jama-Alol and colleagues [3]. These data included women who were sterilised in WA from 1980 onwards so the women in this current study had a minimum sterilisation look back period of 10 years.

#### Index admission and hospital type

For the purposes of this study, index SR was defined as the patient’s first SR procedure at a WA hospital during the study period, and hospital types were categorised as public/tertiary metropolitan, private metropolitan, or rural (both public and private).

#### Statistical analyses

Annual overall and age-group-specific ≤29, 30–39 and 40–49 years incidence rates for SR were calculated for the study sample. The numerator consisted of the numbers of cases of incident SR for the specific calendar year and the denominator was the population at risk, defined as the population of women in the relevant age range who had previously had a sterilisation procedure post-1979, but not a SR. Annual proportions of women who underwent SR procedures were also calculated for each hospital type.

Logistic regression models were also used to estimate odds ratios (ORs) and 95% confidence intervals (CIs) of SR by each hospital type. For the purposes of this analysis, hospital types were categorised as metropolitan private versus all other types combined (metropolitan public/tertiary and rural [public/private]), and the periods 1997–2001 and 2002–2008 were compared with the period 1990–1996 as the reference to evaluate changes over time. Models were fitted with and without adjustment for age group.

Analyses of incidence rates of SR were performed to evaluate the effects of Federal Government policies. After determining the distribution of the data was suitable, Poisson regression models were used to estimate rate ratios (RRs) and 95% CIs that compared rates of SR pre- and post-policy implementation. Two comparisons were modelled. The first compared the rate post-Lifetime Health Cover policy (2001–2008) to the rate prior to its implementation (1990–1999). The second compared the rate following the period of policy implementations (2002–2008) to the rate prior to any of the policy implementations (1990–1996). These models included adjustment for trend and ‘washout’ periods for the year(s) of implementation. Rate comparisons were made for all women, and, in separate models, for women aged ≤29, 30–39 and 40–49 years. Statistical analyses were conducted using SPSS Statistics Software (Version 18) [28] and statistical significance was set at 0.05.

## Results

Characteristics of the study sample are shown in Table 1. Overall, 1868 index SR procedures were performed in WA women aged 15–49 years between 1990 and 2008. The majority of women who underwent SR were aged 30–39 years (65.7%). Mean ± SD age at first hospital admission for SR was 33.3 ± 5.0 years (median: 33, range: 19–49 years).

**Table 1:** Characteristics of women undergoing incident Sterilisation Reversal in WA from 1990 to 2008

Characteristic	Women with sterilisation reversal (*n* = 1868)	
	N	Percent
Age-group (years)		
≤ 19	<5	(0.1)
20–29	435	(23.3)
30–39	1227	(65.7)
40–49	205	(11.0)
Indigenous status		
Indigenous	125	(6.7)
Non-Indigenous	1743	(93.3)
Parity		
0	558	(29.9)
1	529	(28.3)
2	465	(24.9)
3	222	(11.9)
≥ 4	94	(5.0)
Geographical region of birth		
Australia & New Zealand	1354	(72.5)
Asia	50	(2.7)
America	<5	(0.1)
Europe	340	(18.2)
North Africa & Middle East	22	(1.2)
Other Africa	17	(0.9)
Other Oceania	59	(3.2)
Inadequately described (at sea or not stated)	24	(1.3)
Hospital Categories		
Public metropolitan/tertiary	908	(48.6)
Private metropolitan	497	(26.6)
Rural public/private	463	(24.8)

Over 93% of women who underwent SR were non-Indigenous. The majority (72.5%) were born in Australia or New Zealand, followed by Europe (18.2%). Around half of the SR procedures occurred in public metropolitan hospitals (48.6%), while private metropolitan hospitals accounted for 26.6%, and rural hospitals (public/private) accounted for 24.8% of these procedures during the study period.

**Fig. 1:**
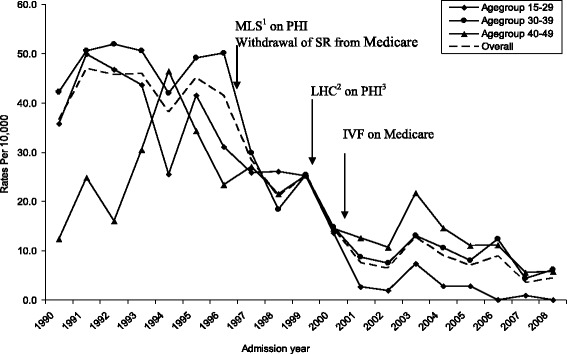
Sterilisation reversal annual specific rates among WA women overall and by age group (1990–2008). ^1^MLS: Medicare Levy Surcharge, ^2^LHC: Lifetime Health Cover, ^3^PHI: Private Health Insurance

**Fig. 2:**
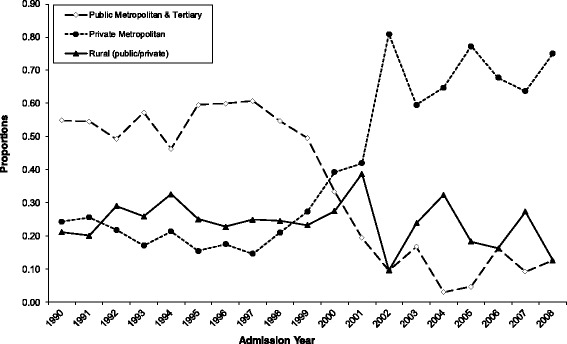
Sterilisation reversal proportions among WA women by hospital type (1990–2008)

Overall, annual rates of SR per 10,000 women declined from a peak of 47.0 in 1991 to a low of 3.6 in 2007 (Fig. 1) (This figure also shows the time points when various policies were implemented). The highest incidence rates for SR were observed among women aged 30–39 years, but this was not the case for every year of the study period (Fig. 1). SR procedures were preformed most frequently at WA public metropolitan hospitals prior to 2000, but subsequently private metropolitan hospitals performed the greatest proportion of these procedures (Fig. 2).

Unadjusted logistic regression modelling showed that the odds of women undergoing SR in private as opposed to all other hospitals were 1.39 times higher (95% CI 1.07–1.81) from 1997 to 2001, increasing to 7.51 times higher (95% CI 5.46–10.31) from 2002 to 2008 compared to the period 1990–1996 (prior to any policy changes)(Table 2).

**Table 2:** Odds of sterilisation reversal in WA metropolitan private versus all other hospitals (1990–2008)

Sterilisation reversal in private versus all other hospitals	Unadjusted OR 95% CI	Adjusted OR 95% CI
Prior to policy changes (1990–1996)^a^		
Policy changes (1997–2001)	1.39 1.07–1.81	1.35 1.03–1.77
Post policy changes (2002–2008)	7.51 5.46–10.31	6.39 4.62–8.84
≤29 years^b^		
30–39 years	2.98 2.17–4.09	2.63 1.90–3.64
40–49 years	7.05 4.72–10.52	5.50 3.63–8.34

*CI* Confidence interval

*OR* Odds ratios

^a^Reference

^b^Reference

The age-group adjusted odds of women undergoing SR in private hospitals were 1.35 times higher (95% CI 1.03–1.77) from 1997 to 2001 and 6.39 times higher (95% CI 4.62–8.84) from 2002 to 2008 compared to the pre-policy implementation period (Table 2). Unadjusted logistic regression modelling showed that the odds of women undergoing SR in private as opposed to all other hospitals were 2.98 times higher (95% CI 2.17–4.09) in the 30–39 age-group and 7.05 times higher (95% CI 4.72–10.52) among the 40–49 age-group compared to the ≤29 age group.

Poisson regression modelling of SR data from 1990 to 2008 showed that, after adjusting for trend, overall incidence rates of SR reduced by 80% after the introduction of Lifetime Health Cover on the PHI in 2000 (RR 0.20; 95% CI 0.17–0.23), and reduced by 83% after introduction of other policies from 2002 to 2008 (RR 0.17; 95% CI 0.15–0.20). Similar reductions in SR incidence rates were seen across age groups (Table 3).

**Table 3:** Rate ratios from Poisson regression models for Sterilisation Reversal among WA Women (1990–2008)

Admission year	Sterilisation Reversal (SR)			
	Overall	≤29 years	30–39 years	40–49 years
Lifetime health cover on PHI (2000)	RR 95% CI	RR 95% CI	RR 95% CI	RR 95% CI
1990-1999^a^				
2001–2008	0.20 0.17–0.23	0.07 0.04–0.10	0.22 0.18–0.25	0.45 0.33–0.62
All health policies on PHI (1997–2001)				
1990-1996^b^				
2002–2008	0.17 0.15–0.20	0.06 0.04–0.09	0.19 0.16–0.22	0.43 0.30–0.62

*RR* Rate ratio

*CI* Confidence interval

*PHI* Private health insurance

^a^Reference

^b^Reference

## Discussion

Using whole-population linked administrative health data, we found a steep decline from 1990 to 2008 in SR rates among WA women overall and within different age-groups. Variation between age-groups was observed, with the highest overall rates of SR in women aged 30–39 years. This is in contrast to findings of a Canadian study which found that younger women had higher SR rates [29]. Our study showed that SR rates decreased among WA women after 1997 when the Australian Federal Government delisted SR from Medicare [19], and the Medicare Levy Surcharge policy was enacted increasing PHI uptake [16, 17]. As both policies were implemented in the same year, it was not possible to differentiate their separate effects in this study. In 2000, Lifetime Health Cover was introduced to further increase PHI uptake [16] and by the end of 2000 43% of the Australian population was covered by PHI [16]. This increase might explain the strong shift of SR procedures to private hospitals after this time. By 2002 the odds of women undergoing SR in private hospitals were 7.5 times greater than in 1990–1996 (prior to policy changes).

SR has significantly higher cumulative pregnancy rates and is more cost-effective than IVF [30], however, in Australia and internationally, IVF has become increasingly available over the last three decades for restoring fertility [12, 31–33]. Since 2001 all ‘medically necessary’ assisted reproductive treatments have been subsidised through Medicare in Australia [12]. However, as data were not available on women who had access to IVF in WA, we could not directly determine their contribution to the observed decrease in SR rates.

The substantial reductions in SR over the observation period are also likely (at least in part) due to the declining sterilisation rates among WA women over the last two decades [3]. Drago and colleagues (2011) suggested that the announcement of the Australia’s ‘Baby Bonus’ policy in 2004 (which provided a financial incentive per child born) increased fertility intentions [20, 21]. Our previous study of WA women, which showed sterilisation rates dropped by 30% after the implementation of this policy, supports this suggestion [3]. However, whether increased fertility intentions after the introduction of the ‘Baby Bonus’ policy [20, 21], would encourage women to restore fertility by SR is much less certain especially given the costs involved with unsubsidised SR which would offset any financial gains under the Baby Bonus scheme. In addition, there are many financial and clinical implications associated with the choice of restoring fertility, particularly for women aged 40 years and older [4, 34].

The limitations of this study include the absence of population-based comparison group data to investigate the influence of reproductive patterns more generally as predictors of SR. In addition, this study lacked IVF data, therefore we were unable to evaluate the effect of IVF on SR rates. Furthermore, although the current study included a minimum 10-year look back, women who were sterilised prior to 1980, or in other countries or states were not included in the ‘at risk’ population denominators.

Future research incorporating more extensive data linkage along these lines combined with qualitative investigation of women’s choices would help to establish a more comprehensive understanding of the factors that influence declining rates of SR among women as seen in this study.

## Conclusions

In summary, this whole-population study is the first to describe trends in the rates of SR in Australia. Rates of SR decreased over the study period, with significant associations between the admission period and SR decline, overall and by age-group. During the study period there was variation between hospital type and the occurrence of SR throughout the policy implementation periods. Odds of women undergoing SR in private hospitals increased post-2000 with rates of SR procedures impacted by Federal Government interventions, such as withdrawal of the Medicare subsidy for SR procedures and the introduction of several PHI policies.

## Abbreviations

CIs: Confidence intervals
HMDC: Hospital morbidity data collection
IVF: Vitro fertilisation
LHC: Lifetime health cover
MLS: Medicare levy surcharge
MNS: Midwives notification system
ORs: Odds ratios
PHI: Private health insurance
RRs: Rate ratios
SR: Sterilisation reversals
WA: Western australia
WADLS: WA data linkage system
